# Patterns and Predictors of Urinary Continence Recovery After Extraperitoneal Single-Port Robot-Assisted Radical Prostatectomy

**DOI:** 10.3390/jcm15072563

**Published:** 2026-03-27

**Authors:** Lorenzo Santodirocco, Luca A. Morgantini, Marwan Alkassis, Jinchun Qi, Simone Crivellaro

**Affiliations:** 1 Department of Urology, University of Illinois at Chicago, Chicago, IL 60607, USA; lmorga5@uic.edu (L.A.M.); qijinchun@hebmu.edu.cn (J.Q.); crivesim@uic.edu (S.C.); 2 Department of Maternal-Infant and Urological Sciences, Sapienza University of Rome, Umberto I Hospital, 00161 Rome, Italy; 3 Department of Urology, Second Hospital of Hebei Medical University, Shijiazhuang 050004, China

**Keywords:** robot-assisted radical prostatectomy, urinary continence, nerve-sparing surgery

## Abstract

**Background/Objectives**: Urinary continence recovery after robot-assisted radical prostatectomy (RARP) follows a progressive trajectory that is often simplified into binary outcomes. Modeling continence recovery as an ordered process may better reflect post-operative functional patterns and identify clinically relevant predictors. **Methods**: We retrospectively analyzed 180 patients undergoing extraperitoneal single-port RARP. At 6 months, continence recovery was classified into three ordered categories: early continence, late continence, and persistent incontinence. Multivariable ordinal logistic regression was used to identify independent predictors of continence recovery. The primary model included nerve-sparing (NS) status, postoperative complications, age, and prostate volume. Sensitivity analyses were performed by sequentially replacing prostate volume with body mass index, surgical case number, or preoperative prostate-specific antigen (PSA). An interaction between NS and age group was also tested. **Results**: NS surgery was the factor most strongly associated with favorable continence recovery (*p* < 0.001), followed by absence of post-operative complications (*p* = 0.003). Younger age and larger prostate volume were also independently associated with improved continence recovery. Sensitivity analyses confirmed the robustness of the primary model, as replacement of prostate volume with body mass index, surgical case number, or PSA did not improve model performance and did not alter the effect of NS surgery. No significant interaction between NS and age group was observed. **Conclusions**: Continence recovery after extraperitoneal RARP is primarily associated with NS surgery and an uncomplicated post-operative course, with age and prostate volume providing additional refinement. Modeling continence as an ordinal outcome offers a clinically meaningful framework for evaluating functional recovery after prostatectomy.

## 1. Introduction

Radical prostatectomy is a standard curative option for clinically localized prostate cancer, and the widespread adoption of robot-assisted radical prostatectomy (RARP) has substantially improved perioperative and functional outcomes compared with open and pure laparoscopic approaches [1,2]. Nevertheless, post-prostatectomy urinary incontinence remains one of the most relevant determinants of post-operative quality of life, with reported continence rates at 12 months ranging from approximately 70% to 95%, depending on the definition of continence and the assessment method used [2,3]. Early urinary incontinence within the first weeks after surgery is particularly frequent, and although most patients ultimately regain satisfactory continence, a non-negligible proportion experience persistent symptoms that may require additional treatment [4].

Multiple patient- and surgery-related factors have been implicated in continence recovery after RARP, including age, comorbidities, prostate volume, and adjuvant therapies, as well as technical aspects such as bladder neck preservation, posterior and anterior reconstruction, and periurethral suspension [3,5]. Among these, preservation of the neurovascular bundles through nerve-sparing (NS) techniques has consistently been associated with improved early and mid-term urinary continence, as well as better erectile function, without compromising oncological control in appropriately selected patients [1,6,7]. Recent systematic reviews and meta-analyses have shown that NS RARP is linked to higher continence rates up to 6–12 months postoperatively compared with non-nerve-sparing procedures, even when different reconstructive strategies are considered [5,6,7].

More recently, single-port (SP) robotic platforms and extraperitoneal approaches have been introduced to reduce invasiveness while maintaining oncologic and functional outcomes comparable to multiport transperitoneal RARP [8]. Early series of SP extraperitoneal RARP have reported favorable perioperative profiles and high early continence rates, suggesting that this technique may facilitate a rapid functional recovery [9,10]. However, data on the patterns and predictors of urinary continence recovery after extraperitoneal SP-RARP remain limited, and most available studies do not model continence as an ordered process that captures the full trajectory from early recovery to persistent incontinence [5,9]. In this context, the present study aims to characterize distinct patterns of urinary continence recovery after extraperitoneal SP-RARP and to identify independent predictors of early, late, and persistent incontinence using an ordinal modeling framework.

## 2. Materials and Methods

### 2.1. Patient Selection

We retrospectively reviewed the records of the first 180 consecutive patients who underwent Single-Port Robot-Assisted Radical Prostatectomy (SP-RARP) performed by a single surgeon between January 2019 and June 2025, corresponding to the initial experience following the introduction of the da Vinci Single-Port robotic platform at our center. Inclusion criteria were patients undergoing extraperitoneal SP-RARP for localized prostate cancer with available continence follow-up at 30 days and 6 months. Patients with incomplete continence data, prior pelvic radiotherapy, salvage prostatectomy, preoperative urinary incontinence, severe lower urinary tract symptoms requiring treatment, or prior prostate or urethral surgery (e.g., transurethral resection of the prostate) were excluded. Baseline urinary function was not systematically quantified using standardized questionnaires (e.g., IPSS or ICIQ) in this cohort and was therefore not available for analysis. The primary surgeon already had >10 years of experience and >100 cases of multi-port RARP. All procedures were performed using an extraperitoneal approach.

### 2.2. Surgical Procedure

The technique for extraperitoneal SP-RARP has been previously described [1]. In brief, patients are positioned in the supine position, and an 18/20Ch Foley catheter is placed to empty the bladder. A 3–4 cm suprapubic incision is made 3 fingerbreadths up from the pubic bone. After incision of the rectus fascia, finger dissection is used to develop the extraperitoneal space. The Da Vinci SP access kit with an 8 mm assistant port is positioned and after insufflation with the valveless AirSeal (ConMed, Utica, NY, USA), the robot is docked and the instrument inserted. For suction and irrigation, the ROSI (Vascular Technology Incorporated, Nashua, NH, USA) is inserted through the assistant port. After dissection of the retropubic extraperitoneal fat of the Retzius space, the prostate and anterior bladder neck are reached.

The endopelvic fascia was incised bilaterally and the dorsal venous complex was controlled. Bladder neck dissection was performed to preserve its anatomy whenever feasible. Posterior dissection allowed identification and transection of the vas deferens and seminal vesicles, followed by careful control of the vascular pedicles.

When oncologically appropriate, a nerve-sparing (NS) approach was adopted, with meticulous dissection of the neurovascular bundles using predominantly blunt techniques and minimal use of thermal energy. Attention was paid to apical dissection and urethral transection to maximize urethral length preservation.

Pelvic lymph node dissection was performed when indicated according to current guidelines. For the reconstruction part, a modified Rocco stitch [11] for posterior reconstruction is employed, using a 3.0 V-Loc™ suture (Covidien, Mansfield, MA, USA); the same suture is used to start the anastomosis from 6 o’clock location in a clockwise fashion. A second 3.0 V-Loc™ (Covidien, Mansfield, MA, USA) suture is employed in a counterclockwise fashion to complete the anastomosis.

### 2.3. Statistical Analysis

The statistical analyses were performed using IBM SPSS Statistics version 30.0.0.0 (IBM Corp., Armonk, NY, USA). Continuous variables were reported as mean and standard deviation (SD) for normally distributed variables, or median and interquartile range (IQR) for non-normally distributed data. Categorical variables were reported as frequencies and percentages. Normality of distribution was assessed using the Shapiro–Wilk test.

Patients were classified into three ordered continence recovery patterns at 6 months:

- Early continence recovery: pad-free status at 30 days and maintained at 6 months.
- Late continence recovery: incontinent at 30 days with recovery within 6 months.
- Persistent incontinence: incontinent at both 30 days and 6 months.

Comparisons across continence groups were performed using the one-way ANOVA or Kruskal–Wallis test for continuous variables and the chi-square or Fisher’s exact test for categorical variables, as appropriate.

The primary outcome of the study was the urinary continence recovery pattern within the first six months after surgery. Continence status was assessed primarily during routine outpatient clinic visits and recorded in the medical chart based on patient self-report. When an in-person visit was not available, information was obtained from documented clinical records of follow-up encounters; no structured telephone interviews were systematically used for outcome assessment. Urinary continence was defined as a complete pad-free status (0 pads per day) without the use of a “security pad”. Pad weight testing was not routinely performed.

Given the ordinal nature of the outcome, multivariable ordinal logistic regression models (proportional odds models) were used to identify independent predictors of continence recovery. The primary multivariable model was deliberately kept parsimonious due to the limited number of patients in the smallest outcome category and included NS status, post-operative complications, age, and prostate volume. Continence recovery was treated as an ordered outcome ranging from persistent incontinence to late continence and early continence, with higher categories representing more favorable recovery patterns. The proportional odds assumption was assessed using the test of parallel lines.

The main exposures of interest included nerve-sparing (NS) status during surgery, post-operative complications occurring within 30 days after surgery, patient age, and prostate volume. Binary variables were coded so that clinically favorable conditions (NS and absence of post-operative complications) were compared against their respective reference categories. Post-operative complications were classified according to the Clavien–Dindo system and, for descriptive purposes, were stratified into minor (grade I–II) and major (grade ≥ III) complications.

NS was included in the model as a binary variable (yes vs. no). Although different grades of nerve preservation (e.g., unilateral vs. bilateral) may provide additional granularity, a dichotomous classification was adopted to maintain model parsimony and avoid overfitting, given the sample size and the distribution of patients across outcome categories.

Follow-up data beyond 6 months were not available for all patients because a proportion of patients were lost to long-term follow-up after the early postoperative period. In particular, last follow-up information was available for 127 of the 180 patients included in the cohort. However, all patients had available continence assessments at both 30 days and 6 months, which represented the predefined time points for the primary outcome analysis. For this reason, the study endpoint was defined at six months, and no imputation procedures were required for the primary analyses.

Sensitivity analyses were performed to assess the robustness of the primary model by sequentially replacing prostate volume with other clinically relevant covariates, including body mass index, surgical case number (log-transformed), and preoperative PSA, while keeping NS status, post-operative complications, and age constant. An additional model tested the interaction between NS and age group (<65 vs. ≥65 years). To address potential confounding by preoperative cancer-risk variables and to strengthen the evidence for the independent association between NS and continence recovery, an additional sensitivity analysis was performed that simultaneously incorporated two key preoperative oncologic markers: preoperative prostate-specific antigen (PSA, continuous, ng/mL) and ISUP grade group (ordinal, categories 1–5, with ISUP grade 5 as the reference category). In this extended model, NS status, post-operative complications, age, and prostate volume were retained as per the primary model. This approach allows assessment of whether the observed association between NS surgery and continence recovery persists after adjustment for preoperative tumor burden and grade, thereby reducing the likelihood of treatment-selection bias driven by oncologic risk stratification.

Results are reported as odds ratios (ORs) with 95% confidence intervals (CIs). A two-sided *p*-value < 0.05 was considered statistically significant.

## 3. Results

### 3.1. Baseline Characteristics by Continence Group

A total of 180 patients were included in the analysis and stratified into three continence recovery groups: early continence (41 patients, 22.8%), late continence (67 patients, 37.2%), and persistent incontinence (72 patients, 40%). Baseline characteristics according to continence recovery patterns are reported in Table 1.

Patients with persistent incontinence were significantly older compared with those achieving early or late continence recovery (mean age 65.0 vs. 61.7 and 64.2 years, respectively; *p* = 0.03). No significant differences were observed among groups in terms of body mass index, previous abdominal surgery, operative time, estimated blood loss, or total opioid consumption on post-operative day 0.

NS surgery was performed more frequently in patients who achieved early and late continence recovery compared with those with persistent incontinence (82.9% and 79.1% vs. 45.8%, respectively; *p* < 0.001). Similarly, post-operative complications within 30 days were significantly more common in patients with persistent incontinence compared with the other groups (*p* < 0.001). Readmission rates showed a borderline significant difference across continence groups (*p* = 0.05), whereas complication severity according to Clavien–Dindo classification did not differ significantly. A detailed breakdown of post-operative complication types according to the continence recovery group is provided in Appendix A (Table A1).

Preoperative oncological characteristics, including ISUP grade, PSA levels, and prostate volume, were comparable among the three groups. In contrast, adverse pathological features were more frequently observed in patients with persistent incontinence, including higher pathological T stage (*p* = 0.003), nodal involvement (*p* < 0.001), and positive surgical margins (*p* = 0.005).

A detailed description of urinary continence status and pad use at different post-operative time points is provided in Appendix A (Table A2).

### 3.2. Multivariable Ordinal Logistic Regression

To account for the ordered nature of continence recovery, an ordinal logistic regression model was constructed. The proportional odds assumption was satisfied (test of parallel lines, *p* > 0.05). In the multivariable model, NS surgery (OR ~4.48), absence of post-operative complications (OR ~2.7), younger age (OR ~0.95 per year), and larger prostate volume (OR ~1.17 per 10 mL) were independently associated with a more favorable continence recovery profile (Table 2). The model showed good overall fit (Nagelkerke R^2^ = 0.23). Although the effect size for prostate volume was modest, its association remained statistically significant after adjustment for the main covariates, suggesting a possible association between prostate size and continence recovery patterns in this cohort.

**Figure 1:**
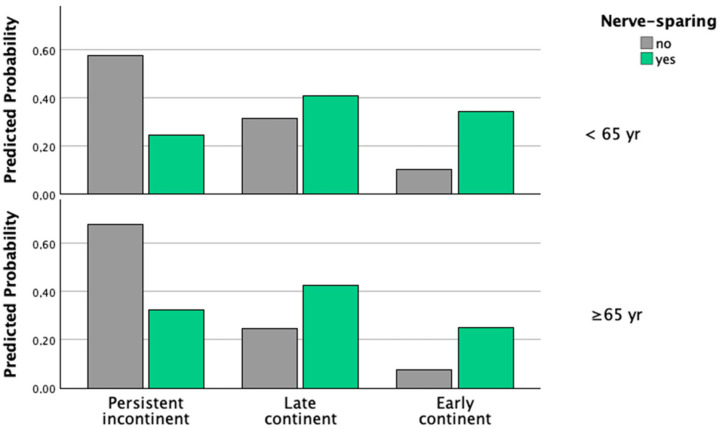
Predicted probabilities of continence recovery according to nerve-sparing status and age group derived from the multivariable ordinal logistic regression model.

BMI, surgical case number, and PSA were not independently associated with continence recovery, and sensitivity analyses replacing prostate volume with these variables did not improve model performance or alter the association between NS surgery and continence recovery (Appendix B, Table A3, Table A4 and Table A5). Likewise, no statistically significant interaction between NS and age group was observed (*p* = 0.81; Appendix B, Table A6). Predicted probability plots derived from the ordinal regression model showed a markedly higher likelihood of early continence recovery in patients undergoing NS surgery, with consistent effects across age groups (Figure 1).

## 4. Discussion

Urinary continence recovery after RARP is reported with substantial variability across studies, largely because definitions (e.g., strict 0-pad vs. “security pad”) and follow-up time points are heterogeneous, which complicates direct cross-study comparisons [3,12].

Our study demonstrates that NS surgery was most strongly associated with favorable continence recovery after extraperitoneal SP-RARP, followed by the absence of post-operative complications, younger age, and larger prostate volume. By modeling continence as an ordered outcome, we captured nuanced recovery patterns not evident in binary assessments, with 22.8% achieving early continence, 37.2% late, and 40% with persistent incontinence at 6 months. The longitudinal distribution of continence status and pad use (Appendix A, Table A2) further supports the progressive nature of functional recovery after extraperitoneal SP-RARP, showing not only increasing pad-free rates over time but also a gradual reduction in incontinence severity among non–pad-free patients. Although the primary endpoint was defined at 6 months, longer-term follow-up data were available for a subset of patients (127/180). At the last available follow-up, 74% of these patients achieved complete continence, suggesting continued recovery beyond the 6-month time point.

These findings align with extensive literature confirming NS as a cornerstone for functional preservation in RARP [5,7,13,14]. In our cohort, NS rates were markedly higher in early/late recovery groups (82.9%/79.1% vs. 45.8% in persistent; *p* < 0.001), mirroring large series where NS independently predicted pad-free status at 3–12 months (OR 2.1–4.5) [6,7,13]. Notably, our extraperitoneal SP approach preserved this benefit without transperitoneal caveats, consistent with early SP-RARP reports of 80–95% continence at 6 months [1,2].

However, NS is not randomly assigned and is inherently influenced by tumor characteristics, oncologic risk, and intraoperative factors. Patients with more favorable disease profiles are more likely to undergo NS procedures, which may introduce treatment-selection bias. Therefore, the observed association between NS and continence recovery should be interpreted with caution and cannot be considered strictly causal. When preoperative ISUP grade group and PSA were simultaneously incorporated into the model (Appendix B, Table A7), NS retained its strong association with favorable continence recovery (OR 4.42, 95% CI 2.43–8.06, *p* < 0.001), with minimal attenuation from the primary model. Neither ISUP grade (*p*-values ranging from 0.43 to 1.00 across categories) nor PSA (OR 1.00, *p* = 0.94) showed independent associations with continence recovery.

In our analysis, post-operative complications emerged as the second strongest predictor, a novel emphasis in SP-RARP contexts [1,15]. However, prior evidence suggests that post-operative morbidity, including early surgical complications such as anastomotic leakage and lymphoceles, can negatively impact early functional outcomes, including urinary continence [16]. Additionally, comprehensive reviews of post-prostatectomy urinary incontinence emphasize the multifactorial nature of functional recovery, involving patient-, surgical-, and perioperative factors [17]. In this context, our findings further suggest that post-operative complications may represent an important and potentially modifiable factor associated with delayed continence recovery.

Age behaved as an independent predictor in our model, and this direction agrees with multiple RARP series in which increasing age is associated with lower odds of early pad-free continence and/or slower time-to-continence recovery [7,12,18].

Although predicted probability plots suggested lower absolute continence recovery rates in older patients (Figure 1), no statistically significant interaction between NS and age group was observed (Appendix B, Table A6). This indicates that while age influences baseline continence recovery, the relative benefit of NS surgery remains consistent across age strata. These findings support the use of NS techniques whenever oncologically appropriate, even in older patients, as age alone does not appear to modify the effect of nerve preservation on continence recovery.

The association we observed for prostate volume should be interpreted against a mixed literature: large registry data (CaPSURE) suggest baseline prostate volume predicts urinary function recovery after radical prostatectomy, with larger glands showing lower continence scores up to 1–2 years [19], whereas other RARP cohorts found prostate weight/volume not independently predictive once age and symptoms are accounted for [12].

For BMI, our null finding is consistent with multivariable RARP analyses where BMI did not independently predict early pad-free status [12], but longer-term evidence remains conflicting because some studies/meta-analytic syntheses report higher 12-month incontinence odds in obese patients [20], while other cohorts show similar continence outcomes between obese and non-obese men when the technique is standardized [21].

Preoperative PSA was not independently associated with continence recovery in our cohort. This finding is consistent with previous RARP series showing that PSA primarily reflects tumor burden and oncologic risk, rather than functional recovery, once age, surgical technique, and NS status are accounted for [11,13].

The lack of an association between surgical case number and continence recovery likely reflects prior surgeon expertise. The adoption of the SP platform preserves the same anatomical landmarks and NS principles, potentially minimizing platform-related learning effects on continence outcomes. As a matter of fact, early functional outcomes reported in initial series of SP-RARP appear favorable even during the early adoption phase [15].

The continence rates observed in our cohort (60% pad-free at 6 months and 74% at last available follow-up among patients with available data) appear lower than those reported in some contemporary RARP series. This difference is likely explained by several factors. First, we adopted a strict definition of continence as complete pad-free status (0 pads per day), without allowing the use of a “security pad,” which is known to yield lower reported continence rates compared with more permissive definitions. Second, our primary endpoint was set at 6 months, whereas many studies report continence outcomes at 12 months or later, when recovery is more complete. Third, our cohort includes the initial institutional experience with the single-port platform, which may reflect early adoption dynamics despite prior surgeon experience. Taken together, these factors likely account for the differences observed when compared with other published series.

Limitations include the retrospective design and tertiary single-center nature, introducing selection bias despite surgeon experience. Although consecutive patients were included to minimize selection bias, unmeasured confounding factors may still influence continence recovery. In particular, standardized measures of baseline urinary function (e.g., IPSS or validated questionnaires), pelvic floor status, and imaging parameters such as membranous urethral length (MUL) [22] were not systematically available and therefore could not be included in the multivariable models. Moreover, the simplified representation of NS as a binary variable may have led to an underestimation of the heterogeneity of the effect of NS on functional outcomes. Finally, incontinence-complication correlations were aggregate, not granular by type, limiting causality insights. Finally, a six-month endpoint may underestimate very late recovery.

## 5. Conclusions

Continence recovery after extraperitoneal SP-RARP was associated with NS surgery and the absence of post-operative complications, with age and prostate volume providing additional refinement. These results support a surgical strategy focused on functional preservation and complication avoidance to optimize post-operative continence outcomes.

## Figures and Tables

**Table 1:** Baseline clinical and pathological characteristics of the study population stratified by continence group.

	Early Continent	Late Continent	Persistent Incontinent	*p*-Value
Number (%)	41 (22.8%)	67 (37.2%)	72 (40%)	
Age (year), mean ± SD	61.7 ± 7.5	64.2 ± 5.8	65 ± 6.6	0.03
BMI (kg/m^2^), median (IQR)	28.9 (26.4–34.8)	27.9 (24.2–32.2)	28.3 (25.2–32.9)	0.21
Previous abdominal surgery, n° (%)				0.36
No	34 (82.9%)	48 (71.6%)	52 (72.2%)	
Yes	7 (17.1%)	19 (28.4%)	20 (27.8%)	
Operative time (min.), mean ± SD	235.9 ± 40	231.3 ± 41.8	237.4 ± 39	0.66
EBL (mL), median (IQR)	100 (50–200)	100 (50–200)	100 (50–100)	0.39
Nerve-sparing, n° (%)				<0.001
No	7 (17.1%)	14 (20.9%)	39 (54.2%)	
Yes	34 (82.9%)	53 (79.1%)	33 (45.8%)	
Post-operative complications, n° (%)				<0.001
No	39 (95.1%)	46 (68.7%)	42 (58.3%)	
Yes	2 (4.9%)	21 (31.3%)	30 (41.7%)	
Readmission, n° (%)				0.05
No	39 (95.1%)	56 (83.6%)	56 (77.8%)	
Yes	2 (4.9%)	11 (16.4%)	16 (22.2%)	
Post-operative complication CD ^1^, n° (%)				0.82
<2	1 (50%)	14 (66.7%)	22 (73.3%)	
≥2	1 (50%)	7 (33.3%)	8 (26.7%)	
Preoperative ISUP grade, n° (%)				0.59
<3	27 (65.9%)	41 (61.2%)	37 (51.4%)	
≥3	14 (34.1%)	26 (38.8%)	35 (48.6%)	
Preoperative PSA (ng/mL), median (IQR)	7.7 (4.7–9.7)	8.5 (6.1–14.6)	8.4 (6–16.7)	0.18
Prostate volume (mL), median (IQR)	44 (37–69)	47 (40–58)	49 (37–58.5)	0.99
Post-operative ISUP grade, n° (%)				0.14
<3	28 (68.3%)	35 (52.2%)	27 (37.5%)	
≥3	13 (31.7%)	32 (47.8%)	45 (62.5%)	
Total opioid dose POD0 (ME), mean ± SD	19.1 ± 7.5	17.9 ± 7.3	19.0 ± 7.3	0.25
Pathological T, n° (%)				0.003
pT2	30 (73.2%)	42 (62.7%)	31 (43.1%)	
pT3a	8 (19.5%)	17 (25.4%)	18 (25%)	
pT3b	3 (7.3%)	8 (11.9%)	23 (31.9%)	
Pathological N, n° (%)				<0.001
pNx	20 (48.8%)	30 (44.8%)	21 (29.2%)	
pN0	21 (51.2%)	33 (49.3%)	34 (47.2%)	
pN1	0	4 (6%)	17 (23.6%)	
Margin status, n° (%)				0.005
R0	27 (65.9%)	47 (70.1%)	32 (44.4%)	
R1	14 (34.1%)	20 (29.9%)	40 (55.6%)	

^1^ Percentages for post-operative complication severity are calculated among patients who experienced complications. IQR, interquartile range; SD, standard deviation; BMI, body mass index; EBL, estimated blood loss; CD, Clavien-Dindo; ISUP, International Society of Urological Pathology; PSA, prostate-specific antigen; ME: milligram equivalent; POD, post-operative day.

**Table 2:** Multivariable ordinal logistic regression analysis of predictors of continence recovery (early, late, persistent) at 6 months after surgery.

	OR	95% CI	*p*-Value
Nerve-sparing (yes vs. no)	4.48	2.32–8.33	<0.001
No post-operative complications (ref: post-operative complications)	2.75	1.40–5.40	0.003
Age (yr.)	0.95	0.92–0.99	0.02
Prostate volume (per 10 mL increase)	1.17	1.03–1.32	0.012

OR: odds ratio; CI: confidence interval. Odds ratios > 1 indicate higher odds of belonging to a more favourable continence recovery category (persistent incontinent → late continent → early continent). For binary variables, the comparison is explicitly indicated in each row (e.g., nerve-sparing: yes vs. no; complications: no complications vs. complications).

## Data Availability

The raw data supporting the conclusions of this article will be made available by the authors upon reasonable request.

## Abbreviations

The following abbreviations are used in this manuscript:

RARP	Robot-Assisted Radical Prostatectomy
NS	Nerve-Sparing
SP	Single-Port
PSA	Prostate-Specific Antigen
BMI	Body Mass Index
ISUP	International Society of Urological Pathology
MUL	Membranous Urethral Length
OR	Odds Ratio
CI	Confidence Interval

## Appendix A

**Table A1:** Post-operative complications profile by continence group.

	Early Continent	Late Continent	Persistent Incontinent
Urine leak, n° (%)	/	5 (23.9%)	4 (13.4%)
UTI and/or sepsis, n° (%)	1 (50%)	2 (9.5%)	7 (23.4%)
Hematuria, n° (%)	/	4 (19%)	9 (30%)
Urinary retention, n° (%)	/	3 (14.3%)	3 (10%)
Lymphocele, n° (%)	/	4 (19%)	5 (16.7%)
Pneumothorax, n° (%)	/	2 (9.5%)	/
Orchitis/epididymitis, n° (%)	/	1 (4.8%)	/
Hernioplasty mesh infection, n° (%)	/	/	1 (3.2%)
Abdominal pain, nausea, n° (%)	1 (50%)	/	1 (3.2%)

UTI, urinary tract infection.

**Table A2:** Urinary continence status and pad use at different post-operative time points.

	30 Days	6 Months	Last-Follow-Up
Continent, n° (%)	41 (22.8%)	108 (60%)	94 (74% ^1^)
Incontinent, n° (%)	139 (77.2%)	72 (40%)	33 (26% ^1^)
Pads/day, n° (%)			
1	66 (47.5%)	33 (45.8%)	15 (45.5%)
2	52 (37.4%)	28 (38.9%)	15 (45.5%)
>2	21 (15.1%)	11 (15.3%)	3 (9%)

^1^ At last follow-up, continence data were available for 127 patients. Continence was defined as a complete pad-free status.

## Appendix B

**Table A3:** Ordinal logistic regression including Body Mass Index.

	OR	95% CI	*p*-Value
Nerve-sparing (yes vs. no)	4.17	2.38–7.14	<0.001
No post-operative complications (ref. post-operative complications)	2.65	1.35–5.22	0.004
Age (year)	0.95	0.90–0.99	0.04
BMI (kg/m^2^)	0.99	0.94–1.05	0.70
Nagelkerke R^2^ = 0.207			

OR: odds ratio; CI: confidence interval. Odds ratios > 1 indicate higher odds of belonging to a more favourable continence recovery category (persistent incontinent → late continent → early continent). For binary variables, the comparison is explicitly indicated in each row (e.g., nerve-sparing: yes vs. no; complications: no complications vs. complications).

**Table A4:** Ordinal logistic regression including the logarithm of the surgical case number.

	OR	95% CI	*p*-Value
Nerve-sparing (yes vs. no)	4.00	2.27–7.14	<0.001
No post-operative complications (ref. post-operative complications)	2.58	1.33–5.24	0.005
Age (per year increase)	0.95	0.91–0.99	0.04
Log. of surgical case number	0.99	0.81–1.29	0.90
Nagelkerke R^2^ = 0.206			

OR, odds ratio; CI, confidence interval. Odds ratios > 1 indicate higher odds of belonging to a more favourable continence recovery category (persistent incontinent → late continent → early continent). For binary variables, the comparison is explicitly indicated in each row (e.g., nerve-sparing: yes vs. no; complications: no complications vs. complications).

**Table A5:** Ordinal logistic regression including the Prostate Specific-Antigen value.

	OR	95% CI	*p*-Value
Nerve-sparing (yes vs. no)	4.17	2.33–7.14	<0.001
No post-operative complications (ref. post-operative complications)	2.62	1.34–5.12	0.004
Age (year)	0.95	0.91–0.99	0.04
PSA (ng/mL)	1.01	0.98–1.04	0.48
Nagelkerke R^2^ = 0.209			

OR, odds ratio; CI, confidence interval; PSA, prostate-specific antigen. Odds ratios > 1 indicate higher odds of belonging to a more favourable continence recovery category (persistent incontinent → late continent → early continent). For binary variables, the comparison is explicitly indicated in each row (e.g., nerve-sparing: yes vs. no; complications: no complications vs. complications).

**Table A6:** Ordinal logistic regression including interaction between nerve-sparing and age group.

	OR	95% CI	*p*-Value
Nerve-sparing (yes vs. no)	4.17	2.17–8.33	<0.001
No post-operative complications (ref. post-operative complications)	2.61	1.33–5.15	0.005
Age group ^1^	0.89	0.46–1.74	0.73
Nerve-sparing × age group	1.17	0.33–4.11	0.81
Prostate volume (mL)	1.02	1.00–1.03	0.019
Nagelkerke R^2^ = 0.214			

^1^ ≥65 yr. vs. <65 yr. OR, odds ratio; CI, confidence interval. Odds ratios > 1 indicate higher odds of belonging to a more favourable continence recovery category (persistent incontinent → late continent → early continent). For binary variables, the comparison is explicitly indicated in each row (e.g., nerve-sparing: yes vs. no; complications: no complications vs. complications).

**Table A7:** Ordinal logistic regression additionally adjusted for preoperative PSA and ISUP grade group.

	OR	95% CI	*p*-Value
Nerve-sparing (yes vs. no)	4.42	2.43–8.06	<0.001
No post-operative complications (ref. post-operative complications)	2.58	1.30–5.12	0.002
Age (year)	0.94	0.90–0.98	0.01
PSA (ng/mL)	1.00	0.97–1.03	0.94
Prostate volume (per 10 mL increase)	1.18	1.04–1.33	0.013
ISUP GG 1	1.00	0.22–4.61	1.00
ISUP GG 2	1.43	0.35–5.87	0.62
ISUP GG 3	1.80	0.42–6.14	0.43
ISUP GG 4	1.16	0.28–4.91	0.84
ISUP GG 5	/	/	/
Nagelkerke R^2^ = 0.242			

OR, odds ratio; CI, confidence interval, PSA, prostate-specific antigen; ISUP, International Society of Urological Pathology; GG, grade group. Odds ratios > 1 indicate higher odds of belonging to a more favorable continence recovery category (persistent incontinent → late continent → early continent). For binary variables, the comparison is explicitly indicated in each row (e.g., nerve-sparing: yes vs. no; complications: no complications vs. complications). ISUP grade group 5 serves as the reference category.
